# Role of FGFR2b expression and signaling in keratinocyte differentiation: sequential involvement of PKCδ and PKCα

**DOI:** 10.1038/s41419-018-0509-x

**Published:** 2018-05-11

**Authors:** Benedetta Rosato, Danilo Ranieri, Monica Nanni, Maria Rosaria Torrisi, Francesca Belleudi

**Affiliations:** 1 Department of Clinical and Molecular Medicine,Laboratory affiliated to Istituto Pasteur Italia—Fondazione Cenci Bolognetti, Sapienza University of Rome, Roma, Italy; 2 S. Andrea University Hospital, Rome, Italy

## Abstract

The tumor suppressor epithelial isoform of the fibroblast growth factor receptor 2 (FGFR2b) induces human keratinocyte early differentiation. Moreover, protein kinases C (PKCs) are known to regulate the differentiation program in several cellular contexts, including keratinocytes. Therefore, in this paper we propose to clarify if FGFR2b could play a role also in the late steps of keratinocyte differentiation and to assess if this receptor-induced process would sequentially involve PKCδ and PKCα isoforms. Immunofluorescence, biochemical, and molecular approaches, performed on 2D cultures or 3D organotypic rafts of human keratinocytes overexpressing FGFR2b by stable transduction, showed that receptor signaling induced the precocious onset and an accelerated progression of keratinocyte differentiation, indicating that FGFR2b is a crucial regulator of the entire program of keratinocyte differentiation. In addition, the use of specific inhibitors and gene silencing approaches through specific siRNA demonstrated that PKCδ controls the onset of FGFR2b-triggered differentiation, while PKCα plays a role restricted to the terminal stages of the process. Molecular analysis revealed that the two PKC isoforms sequentially act via induction of KLF4 and DLX3, two transcription factors linked by negative loops to p63, suggesting that p63 would represent the hub molecule at the crossroad of an intricate signaling network downstream FGFR2b, involving multiple PKC-induced transcription factors.

## Introduction

The family of fibroblast growth factor receptors (FGFRs) includes four transmembrane receptor tyrosine kinases (FGFR1–4) involved in the regulation of crucial biological processes, such as proliferation, migration, survival, and differentiation^1,2^. Alternative splicing of the extracellular IgIII loop in FGFR1-3 generates epithelial FGFRb and mesenchymal FGFRc isoforms, determining ligand specificity^1^. Deregulation of the expression and signaling of FGFRs is well known to play oncogenic roles^3,4^. In the case of FGFR2, altered isoform switching and aberrant expression of the mesenchymal FGFR2c isoform in epithelial cells induce epithelial-mesenchymal transition (EMT)^5,6^ and are involved in cancerogenesis^7,8^, while the epithelial FGFR2b variant appears to exert tumor suppressive functions^9,10^.

In agreement with the knowledge of the key role played by FGFR2b in the regulation of skin homeostasis^11–13^, studies from our group have demonstrated that FGFR2b is up-regulated in epidermal suprabasal layers^14^ and is able to induce early differentiation in normal human keratinocytes^15^. We have also suggested that FGFR2b exerts its differentiative function via the repression of p63^16^, a transcription factor whose down-modulation represents the main molecular mechanism known to drive early differentiation of stratified epithelia^17^. Although the role of FGFR2b in early stages of keratinocyte differentiation has been deeply investigated, its involvement in the later steps of the process still remains debated^18–20^ and to be clarified.

With the aim to analyze the possible signaling candidates for the regulation of FGFR2b-mediated early differentiation, we have previously demonstrated the involvement of the PI3K/AKT pathway^15^. However, the keratinocyte differentiation is a complex program implying several steps of coordinated molecular events driven by an intricate signaling network; therefore, to verify if additional pathways downstream FGFR2b could participate to such network, we focused our attention on protein kinases C (PKCs) and in particular to the PKCδ and PKCα isoforms. In fact, these signaling substrates are involved in FGFR2-mediated differentiation in the bone context^21,22^ and it has been proposed that PKCδ and PKCα could regulate keratinocyte differentiation^23^, during which they appear to exert temporally and spatially distinct roles.

A function in the onset of keratinocyte differentiation has been proposed for PKCδ^23–29^, which is expressed starting from the basal layer of epidermis where, upon activation, causes hemidesmosomal disassembly and basal cell migration upward into the suprabasal layers^30,31^. At the level of transcriptional control, PKCδ induces the early differentiation marker desmoglein-1 (DSG1)^26^. Moreover, recent studies highlighted that PKCδ, through the induction of the transcription factor Kruppel-like factor 4 (KLF4), up-modulates p21 and the intermediate differentiation marker involucrin (INV)^32^ triggering cell cycle arrest and differentiation, respectively^28,29^.

On the other hand, a role in epidermal late differentiation has been proposed for PKCα. In fact, PKCα expression is restricted to epidermal suprabasal layers^23^ and its inhibition or depletion represses the expression of the late differentiation markers filaggrin (FIL) and loricrin (LOR)^32^, while the early/intermediate differentiation markers keratin 1 (K1), K10, and INV^32^ appear substantially unaffected^25,27,33–36^. Distal-less homeobox 3 (DLX3) could be the molecular player acting downstream PKCα in keratinocyte differentiation:^36^ this transcription factor is expressed in the suprabasal layers and is involved in the induction of FIL and LOR, as well as in the epidermal barrier formation^37–39^.

Based on these assumptions, here we propose to clarify if FGFR2b could play a general role in the control of the entire program of keratinocyte differentiation and to assess if the possible function of FGFR2b in the different steps of the process might be regulated by a signaling network sequentially involving PKCδ and PKCα isoforms and their respective downstream transcription factors.

## Results

### FGFR2b controls not only the early, but also the late steps of keratinocyte differentiation

**Fig. 1:**
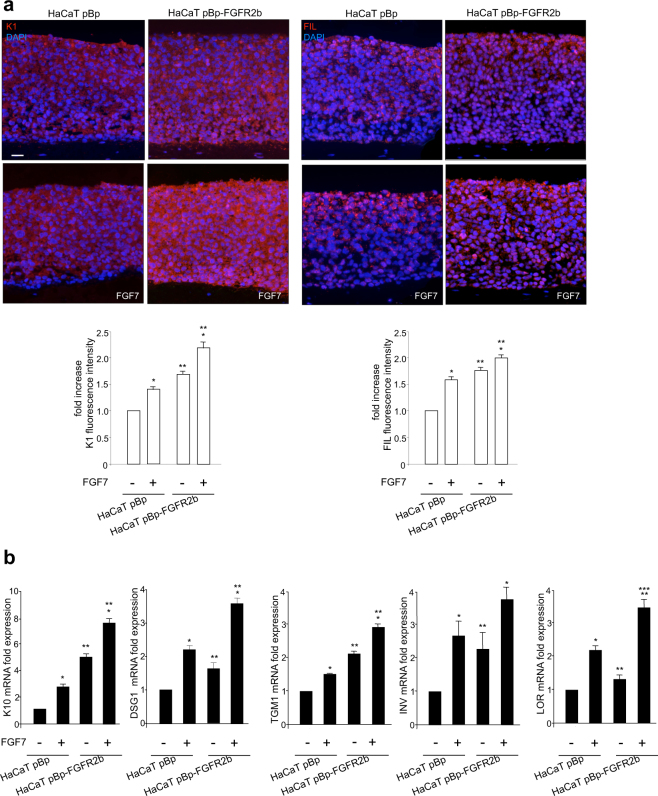
FGFR2b expression and signaling play a positive role on the entire program of keratinocyte differentiation. Organotypic skin equivalents of HaCaT pBp and pBp-FGFR2b clones, prepared as reported in Materials and methods section, were grown in complete medium and left untreated or stimulated with FGF7 for the last 4 days. **a** Quantitative immunofluorescence analysis shows that, in pBp rafts, K1 staining is visible in all suprabasal layers and it is increased by FGF7 stimulation. In HaCaT pBp-FGFR2b raft K1 staining is enhanced and detectable already in the basal layer. Parallel evaluation of FIL expression shows that the its granular staining appears confined in the uppermost layers of pBp skin equivalents and it is significantly increased by FGF7 stimulation. FGFR2b overexpression induces a general enhancement of the staining and its appreciable appearance starting from lower layers of the rafts. Quantitative analysis of the fluorescence intensity was performed as described in Materials and methods and results sections are expressed as fold increase respect to pBp values ± SE. Student’s *t* test was performed and significance levels have been defined as *p* < 0.05: *p < 0.0001 vs the corresponding FGF7-unstimulated cells; **p < 0.0001 vs the corresponding pBp cells. Bar: 25 μm. **b** Real time RT-PCR analysis shows that mRNA expression of the early (K10 and DSG1), intermediate (TGM1 and INV) and late differentiation (LOR) markers are significantly increased upon FGF7 stimulation, particularly in pBp-FGFR2b rafts. Results are expressed as mean values ± SD. Student’s *t* test was performed and significance levels have been defined as *p* < 0.05: *p < 0.05, and ***p < 0.005 vs the corresponding FGF7-unstimulated cells; **p < 0.05 vs the corresponding pBp cells

Studies from our group have demonstrated that FGFR2b expression and activation promote keratinocyte early differentiation^15,16^. Here, in order to investigate if FGFR2b might also control the later stages of differentiation, we first analyzed the impact of the receptor signaling on the expression and distribution throughout the epidermal layers of two distinct markers of the most early or the most late differentiation steps: the early cytokeratin K1 and the terminal differentiation FIL. For this purpose we reproduced in vitro the keratinocyte differentiation program occurring in vivo, using 3D organotypic skin equivalents prepared with human HaCaT keratinocytes^40^ stably transduced with pBp-FGFR2b retroviral constructs or with empty pBp vector as negative control^6^. In fact, through this model of receptor-overexpressing cells we are able to force the system toward the differentiated phenotype. The organotypic cultures were left untreated or stimulated with FGF7 as reported in Materials and methods section. Quantitative immunofluorescence analysis showed that, in pBp rafts, the K1 signal was visible in all suprabasal layers (Fig. 1a, left panels) and increased by FGF7 stimulation (Fig. 1a, left panels), as expected^15^. Interestingly, HaCaT pBp-FGFR2b rafts showed, besides the enhancement of K1 staining (Fig. 1a, left panels), its appearance already in the basal layer (Fig. 1a, left panels), suggesting the precocious onset of early differentiation. Parallel evaluation of FIL expression showed that, as expected^32^, its granular staining appeared confined in the uppermost layers of pBp skin equivalents (Fig. 1a, right panels) and it was significantly increased by FGF7 stimulation (Fig. 1a, right panels). In addition, FGFR2b overexpression induced a general enhancement of this staining and its appreciable appearance starting from lower layers of the rafts (Fig. 1a, right panels), suggesting an accelerated progression also throughout the terminal steps of differentiation. As previously reported^41^, the higher staining of the analyzed markers in pBp-FGFR2b untreated organotypic cultures respect to the corresponding unstimulated pBp rafts would be ascribed to the already acquired enhanced differentiated phenotype of the FGFR2b clones during their growth in complete medium before starvation. Our observations were also validated at molecular level by real time RT-PCR analysis, demonstrating that the expression of early (K10 and DSG1), intermediate (INV, transglutaminase-1: TGM1) and late differentiation marker (LOR) genes were significantly increased in response to FGF7 and particularly in cells overexpressing FGFR2b (Fig. 1b). Thus, FGFR2b and its signaling appear to play a positive role on the entire program of keratinocyte differentiation.

**Fig. 2:**
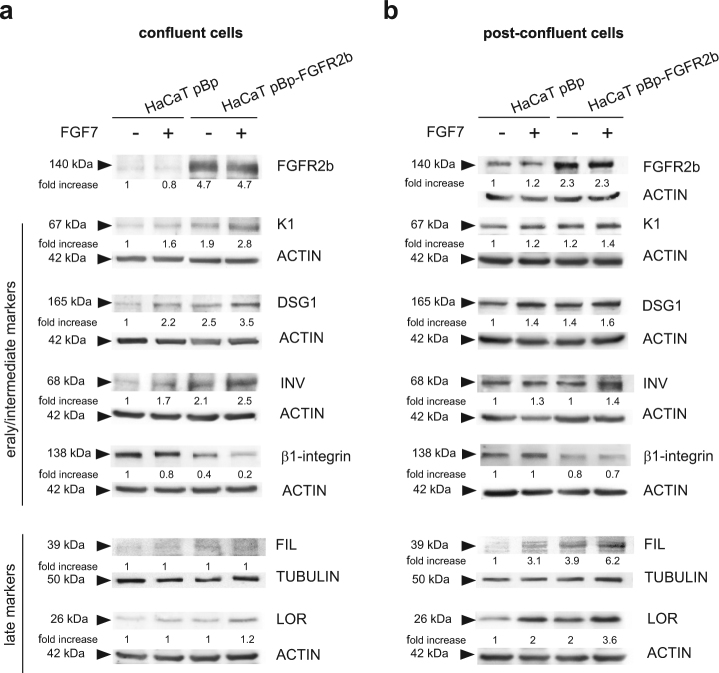
FGFR2b has a sequential role in distinct steps of the keratinocyte differentiation. HaCaT pBp and HaCaT pBp-FGFR2b clones grown up to confluence or post-confluence were left untreated or stimulated with FGF7 as reported in Materials and methods section. **a** Western blot analysis shows that in confluent conditions the levels of K1, DSG1, and INV are increased upon FGF7 stimulation particularly in pBp-FGFR2b cells, while no appreciable levels of FIL and LOR are visible in these cells. In addition, the expression of β1-integrin appears strongly decreased in HaCaT pBp-FGFR2b in response to ligand stimulation. **b** In post-confluence conditions all the early/intermediate differentiation markers appear increased making their FGF7-induced modulation less evident compared to the corresponding confluent cells. In contrast, FIL and LOR are detectable and they appear up-regulated in response to FGF7 stimulation, particularly in cells overexpressing FGFR2b. The equal loading was assessed with anti-ACTIN and anti-TUBULIN antibodies. For densitometric analysis the values from a representative of three independent experiments are reported

In order to analyze in detail this role, we “dissected” in vitro the process using HaCaT pBp and HaCaT pBp-FGFR2b cultures grown until confluence, the step that precedes the shift from the basal to suprabasal layer, or up to post-confluence, the step that mimics the late differentiation and stratification^40^. Cells were then left untreated or stimulated with FGF7, as reported in Materials and methods section, and Western blot analysis was performed to estimate the expression of a wide selection of differentiation markers. Results showed that, in confluent conditions, a clear up-regulation of the early differentiation markers K1 and DSG1 and of the intermediate differentiation marker INV was induced by FGF7, particularly in pBp-FGFR2b cells (Fig. 2a). In addition, an opposite behavior was observed for the basal marker β1-integrin^42^, whose expression appeared strongly decreased in HaCaT pBp-FGFR2b in response to ligand stimulation (Fig. 2a). Finally, in agreement with their similarities with basal/differentiating keratinocytes, no appreciable levels of the late differentiation marker FIL and LOR were found in all confluent HaCaT clones (Fig. 2a). On the other hand, the shift to post-confluence strongly increased the expression of all the differentiation markers (Fig. 2b), making their FGF7-induced modulation less evident compared to the corresponding confluent cells (Fig. 2b). Moreover, as already observed in 3D organotypic cultures, pBp-FGFR2b cells showed a higher differentiated phenotype compared to pBp cultures also in the absence of FGF7 stimulation, which can be ascribed to the enhanced differentiation acquired during the growth in complete medium before starvation. The observed progressive modulation in response to FGF7 of early/intermediate and LORs in confluent and post-confluent cultures of HaCaT clones, respectively, was also confirmed at the mRNA transcript level (Supplementary Figure S1).

### PKCδ and PKCα signaling play sequential roles in FGFR2b-triggered keratinocyte differentiation

Searching for the possible downstream candidates that could be alternatively involved in FGFR2b-mediated early or late differentiation, we focused our attention on PKCδ and PKCα isoforms, since they have been proposed to exert distinct roles in keratinocyte differentiation^23^.

**Fig. 3:**
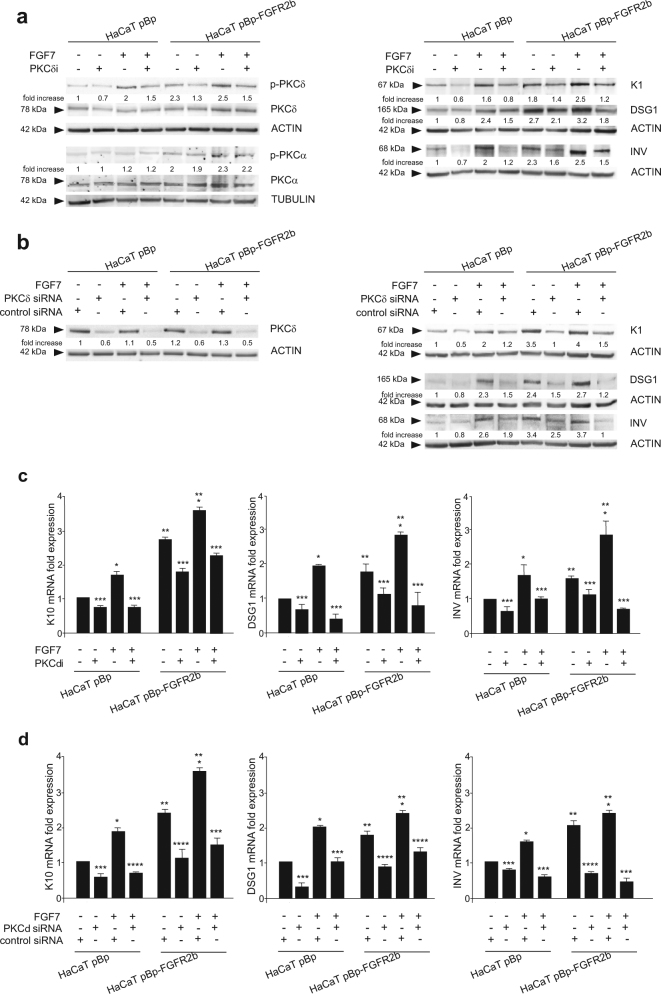
PKCδ controls the onset of FGFR2b-triggered keratinocyte differentiation. Confluent HaCaT pBp and HaCaT pBp-FGFR2b clones were left untreated or stimulated with FGF7 in presence or not of the PKCδ inhibitor as reported in Materials and methods section (**a**, **c**). Alternatively, HaCaT clones were transfected with PKCδ siRNA or with an unrelated siRNA as control, and then left untreated or stimulated with FGF7 (**b**, **d**). **a** Western blot analysis confirms that PKCδ inhibitor interferes with the basal, as well as the FGF7-induced, phosphorylation of PKCδ at its autophosphorylation site Ser645 but not with that of PKCα at its autophosphorylation site Ser657 (left panel). PKCδ inhibition induces a repressive effect on the K1, DSG1, and INV increase induced by FGF7, which is particularly evident in FGFR2b overexpressing clones (right panel). **b** Western blot analysis confirms that PKCδ silencing specifically dampened the basal, as well as the FGF7-induced, PKCδ protein level but not that of PKCα (left panel). PKCδ silencing also strongly counteracts the increase of K1, DSG1, and INV in response to FGF7 (right panel). The equal loading was assessed with anti-PKCδ or anti-PKCα and anti-ACTIN or anti-TUBULIN antibodies. For densitometric analysis the values from a representative of three independent experiments are reported. **c**, **d** Real time RT-PCR analysis confirms that PKCδ inhibition **(c)** or depletion **(d)** decreases FGF7-induced, as well as basal mRNA transcript levels, of K10 DSG1 and INV. Results are expressed as mean values ± SD. Student’s *t* test was performed and significance levels have been defined as above: **c** *p < 0.05 vs the corresponding FGF7-unstimulated cells; **p < 0.05 vs the corresponding pBp cells; ***p < 0.05 vs the corresponding PKCδ inhibitor-untreated cells; **d** *p < 0.05 vs the corresponding FGF7-unstimulated cells; **p < 0.05 vs the corresponding pBp cells; ***p < 0.05 and ****p < 0.005 vs the corresponding control siRNA cells

Since PKCδ isoform has been proposed to be specifically implicated in the onset of the process^23–29^, we first investigated its contribution in FGFR2b-induced early differentiation taking advantage of the specific inhibitor rottlerin (PKCδi), previously used to demonstrate the involvement of PKCδ in FGF/FGFR signaling pathways^21^, as well as in FGFR2b-triggered autophagy^43^. The inhibitor efficiency was confirmed testing its ability to decrease the basal, as well as the FGF7-induced, phosphorylation of PKCδ at the autophosphorylation site Serine 645^44^ (Fig. 3a, left panel), but not that of PKCα at the autophosphorylation site Serine 657^45^ (Fig. 3a, left panel). Then we analyzed the impact of this inhibitor on the modulation of early/intermediate differentiation markers induced by FGFR2b and its signaling in confluent cultures. Western blot analysis clearly showed that PKCδi exerted a strong repressive effect on the K1, DSG1, and INV increase induced by FGF7, which was particularly evident in FGFR2b overexpressing clones (Fig. 3a, right panel). Indeed, in agreement with the proposed general role of PKCδ in keratinocyte early differentiation^23^, this inhibitory effect, even if less pronounced, was also detectable in cells not stimulated with FGF7 (Fig. 3a, right panel). However, to exclude the possibility of the establishment of an autocrine signaling due to endogenous production of FGF7, which could explain the phenotype displayed by unstimulated FGFR2b clones, we performed real time RT-PCR analysis: FGF7 mRNA was undetectable in both HaCaT clones also after PKC signaling shut-off (data not shown). To unequivocally demonstrate the role of PKCδ on early differentiation, we performed its specific depletion by siRNA transfection. To this aim, HaCaT clones were transfected with PKCδ siRNA or with an unrelated siRNA as control. The efficiency of protein depletion was verified through Western blot analysis (Fig. 3b, left panel). Transfected cells were grown until confluence and left untreated or stimulated with FGF7 as above. Western blot analysis showed that PKCδ silencing strongly counteracted the increase of K1, DSG1, and INV in response to FGF7 (Fig. 3b, right panel). In addition, similarly to what observed after the pharmacological inhibition, PKCδ depletion appeared to slightly affect also the basal level of the markers (Fig. 3b, right panel), confirming its general role in the control of early differentiation.

These findings were also validated at the mRNA transcript level by real time RT-PCR analysis, confirming that, in both HaCaT pBp and pBp-FGFR2b confluent cultures, the block of PKCδ activity by PKCδi (Fig. 3c) or protein depletion by specific siRNA (Fig. 3d) strongly decreased FGF7-induced, as well as basal expression, of K10, DSG1, and INV. The results obtained were strengthened by quantitative immunofluorescence analysis (Supplementary Figure S2, left and right panels), in which we introduced also the inhibitor of AKT (AKTi)^15,43,46^, in order to interfere with the additional FGFR2b downstream pathway previously identified by us as involved in keratinocyte early differentiation^15^, or the MEK1/2 inhibitor (MEK1/2i), as negative control, since we have recently observed that this pathway did not appear to be involved in the process (Nanni et al., manuscript in preparation). In fact, while the role played by AKT signaling appears to be restricted to some aspects of FGFR2b-induced early steps of the process, PKCδ emerges as hub signaling downstream FGFR2b regulating the entire early differentiation.

**Fig. 4:**
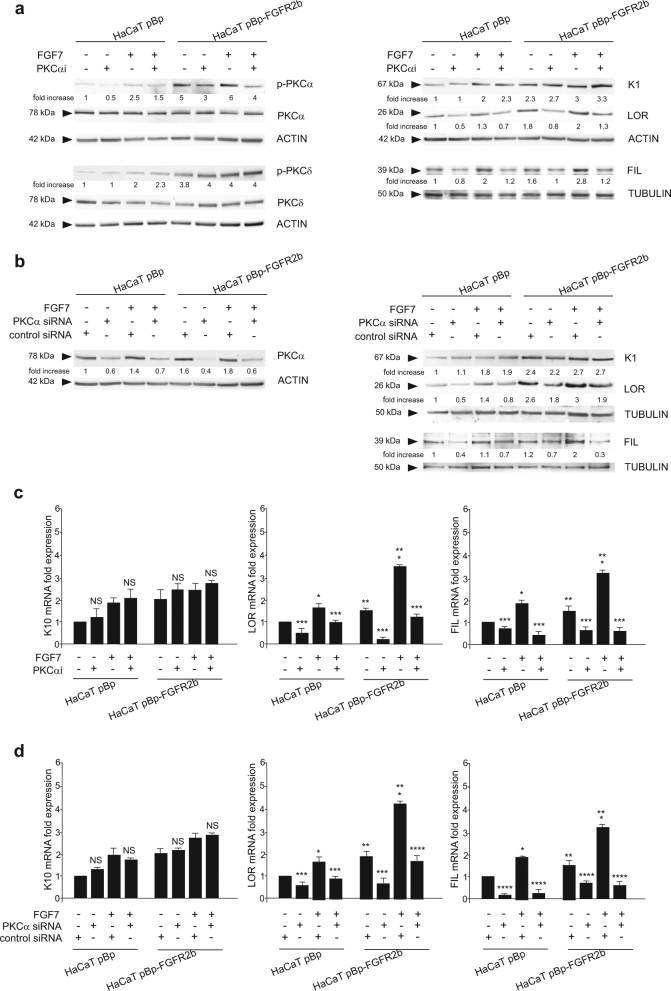
PKCα is required for FGFR2b-induced terminal stages of keratinocyte differentiation. Post-confluent HaCaT pBp and HaCaT pBp-FGFR2b cells were left untreated or stimulated with FGF7 in presence or not of the PKCα inhibitor as reported in Materials and methods section (**a**, **c**). Alternatively, HaCaT clones were transfected with PKCα siRNA or with an unrelated siRNA as control, transfected cells were grown until post-confluence and left untreated or stimulated with FGF7 as above (**b**, **d**). **a** Western blot analysis shows that PKCα inhibitor decreases the basal, as well as the FGF7-induced, PKCα phosphorylation, but not that of PKCδ phosphorylation (left panel). PKCα inhibitor does not interfere with the K1 expression, but it displays a repressive effect on the FGF7-induced up-modulation of LOR and FIL (right panel). **b** Western blot analysis confirms that PKCα silencing specifically dampened the basal, as well as the FGF7-induced, PKCα protein level but not that of PKCδ (left panel). PKCα silencing also strongly counteracts the increase of LOR and FIL but not that of K1 in response to FGF7 (right panel). The equal loading was assessed with anti-PKCδ or anti-PKCα and anti-ACTIN or anti-TUBULIN antibodies. For densitometric analysis the values from a representative of three independent experiments are reported. **c**, **d** Real time RT-PCR analysis confirms that PKCα inhibition (**c**) or depletion (**d**) decreases FGF7-induced, as well as basal mRNA transcript levels, of LOR and FIL but it does not affect K10 expression. Results are expressed as mean values ± SD. Student’s *t* test was performed and significance levels have been defined as above: *p < 0.05 vs the corresponding FGF7-unstimulated cells; **p < 0.05 vs the corresponding pBp cells; NS and ***p < 0.05 vs the corresponding PKCα inhibitor-untreated cells; **d** *p < 0.05 vs the corresponding FGF7-unstimulated cells; **p < 0.05 vs the corresponding pBp cells; NS, ***p < 0.05 and ****p < 0.005 vs the corresponding control siRNA cells

Because PKCα isoform has been proposed to be a signaling substrate crucial for the later steps of keratinocyte differentiation^23,25,27,33–36^, we investigated its possible contribution in FGFR2b-induced terminal steps of the process. To this aim the effects of PKCα inhibition were analyzed in post-confluent cultures of HaCaT clones using the specific inhibitor Go6976 (PKCαi)^36^. The efficiency of PKCαi was assessed by Western blot analysis, performed in both HaCaT pBp and pBp-FGFR2b cultures, showing a decrease of the basal, as well as the FGF7-induced PKCα phosphorylation at the autophosphorylation site Serine 657^45^ (Fig. 4a, left panel), but not that of PKCδ at the autophosphorylation site Serine 645^44^ (Fig. 4a, left panel). Then we investigated if PKCαi could affect FGFR2b-induced differentiation. Western blot analysis highlighted that PKCαi did not interfere with the expression of the early differentiation marker K1 (Fig. 4a, right panel), but it displayed a strong repressive effect on the FGF7-induced up-modulation of the late differentiation markers LOR and FIL (Fig. 4a, right panel). Moreover, also the basal level of LOR and FIL markers expressed in cells not stimulated with FGF7 appeared weakly dampened by PKCαi, confirming the general role of PKCα in keratinocyte differentiation^23^. Similar results were obtained by specific siRNA approaches. The efficiency of protein depletion was first confirmed by Western blot analysis (Fig. 4b, left panel). Then, the effects of PKCα silencing showed repression of the late differentiation markers LOR and FIL (Fig. 4b, right panel), but not of the early differentiation marker K1 (Fig. 4b, right panel) in both pBp and pBp-FGFR2b clones. The inhibitory effect of either PKCα pharmacological inhibition (Fig. 4c) and protein depletion (Fig. 4d) was also confirmed at the mRNA transcript level by real time RT-PCR analysis. Thus, also PKCα appears a pivotal player in FGFR2b-triggered keratinocyte differentiation; however, differently from PKCδ, its role appears to be specifically restricted to the late stages of the process.

**Fig. 5:**
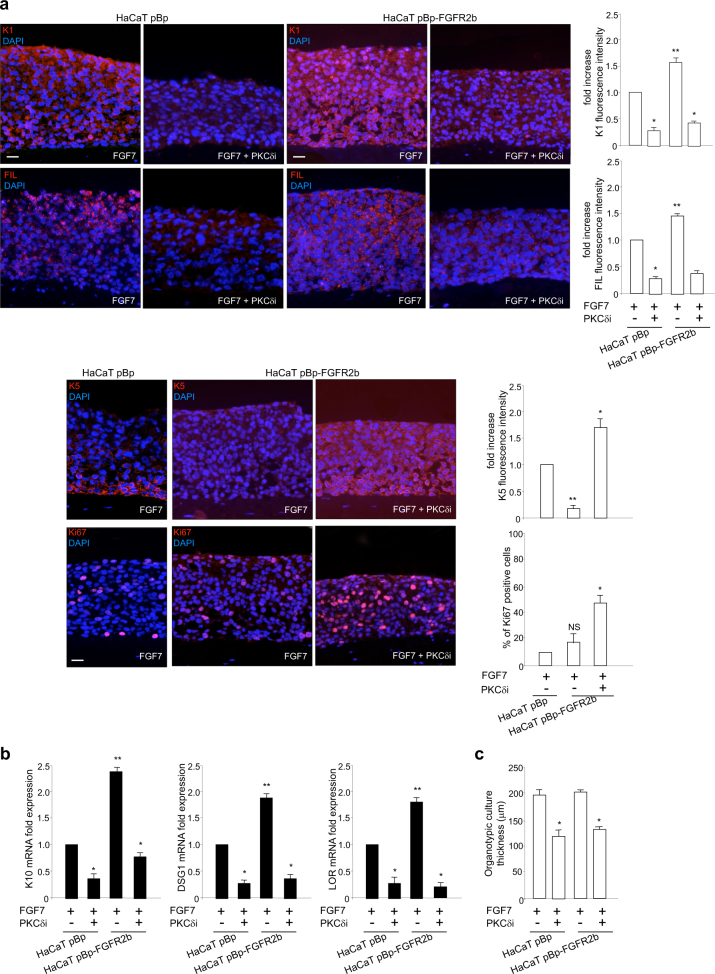
PKCδ signaling shut-off blocks the entire differentiation program and induces defective stratification in keratinocytes. Organotypic skin equivalents of HaCaT pBp and pBp-FGFR2b clones, prepared as reported in Materials and methods section, were lifted to air–liquid interface in the presence or not of PKCδ inhibitor and finally stimulated with FGF7 as above. **a** Quantitative immunofluorescence analysis shows that K1 and FIL staining, visible in both HaCaT pBp and HaCaT pBp-FGFR2b rafts stimulated with FGF7, appears overall impaired by the presence of PKCδ inhibitor (upper panels). In addition, K5 signal, visible in the basal layer of pBp rafts, disappears in pBp-FGFR2b skin equivalents while it is recovered and distributed all over the pBp-FGFR2b rafts by the presence of PKCδ inhibitor (lower panels). Parallel evaluation of the nuclear proliferation marker Ki67 reveals the presence of few positive cells distributed in the basal layer and randomly throughout the suprabasal layers in pBp rafts and FGFR2b rafts stimulated with FGF7 (lower panels). In pBp-FGFR2b raft grown in the presence of PKCδi, several cells intensely stained for Ki67 marker are visible in all the layers (lower panels). Quantitative analysis of the fluorescence intensity and of the percentage of Ki67 positive cells were performed as described in Materials and methods and results sections are expressed as fold increase respect to pBp values ± SE. Student’s *t* test was performed and significance levels have been defined as above: *p < 0.0001 vs the corresponding PKCδ inhibitor-untreated cells; NS and **p < 0.0001 vs the corresponding pBp cells. Bar: 25 μm. **b** Real time RT-PCR analysis confirms that, in pBp and pBp-FGFR2b rafts stimulated with FGF7, the expression of K10, DSG1, and LOR is significantly repressed by PKCδ inhibitor. Results are expressed as mean values ± SD. Student’s *t* test was performed and significance levels have been defined as above: *p < 0.05 vs PKCδ inhibitor-untreated cells; **p < 0.05 vs the corresponding pBp cells. **c** Quantitative evaluation of the raft thickness shows that the layers of pBp and pBp-FGFR2b skin equivalents are significantly reduced by the presence of PKCδ inhibitor. Quantitative analysis of the raft thickness was expressed as µm mean ± SD from three independent experiments. Student’s *t* test was performed and significance levels have been defined as *p* < 0.05: *p < 0.0001 vs the corresponding PKCδ inhibitor-untreated cells

Then, in order to analyze the effect of PKCδ inhibition on the ability of keratinocytes to progress throughout the entire differentiation program and stratify in response to FGFR2b activation, we again took advantage of the use of the more complete model of organotypic cultures. HaCaT pBp and HaCaT pBp-FGFR2b rafts were lifted to air–liquid interface in the presence or not of PKCδi and finally stimulated with FGF7, as above. Quantitative immunofluorescence analysis showed that the K1 and FIL staining, visible in both HaCaT pBp and HaCaT pBp-FGFR2b rafts stimulated with FGF7 (Fig. 5a, upper panels), appeared overall dampened by the presence of PKCδi, suggesting that the entire differentiation process induced by FGFR2b signaling was arrested by the sustained inhibition of PKCδ (Fig. 5a, upper panels). In addition, the immunostaining of K5 revealed that the canonical distribution in the basal layer of this undifferentiated/proliferating cell marker^32^, visible in pBp control rafts (Fig. 5a, lower panels), disappeared in pBp-FGFR2b skin equivalents (Fig. 5a, lower panels), confirming the precocious onset of differentiation. However, in the presence of PKCδi, K5 staining appeared recovered and distributed all over the rafts (Fig. 5a, lower panels). Moreover, the parallel evaluation of Ki67 staining in both pBp and pBp-FGFR2b rafts stimulated with FGF7 revealed few positive cells distributed in the basal layer (Fig. 5a, lower panels); the presence of some stained cells also in the suprabasal layers (Fig. 5a, lower panels) is in agreement with the previously described ability of FGF7 to also sustain the residual proliferative activity of suprabasal keratinocytes^14^. More interestingly, in pBp-FGFR2b raft grown in the presence of PKCδi, several cells intensely stained for Ki67 marker appeared homogeneously distributed in all the layers (Fig. 5a, lower panels), suggesting sustained cell proliferation. These findings strongly indicated that, as a consequence of PKCδ signaling shut-off, keratinocytes are defective in the ability to undergo the differentiation program maintaining a basal/proliferative phenotype. Real time RT-PCR analysis confirmed that the expression of both early and LORs was significantly repressed by PKCδi (Fig. 5b). Finally, the quantitative evaluation of the raft thickness revealed that, in pBp and pBp-FGFR2b skin equivalents, the layers were significantly reduced by PKCδi (Fig. 5c), further indicating that inhibition of PKCδ induces impairment of all steps of FGFR2b-triggered differentiation, which in turn results in defective stratification.

### The transcription factors KLF4 and DLX3, acting downstream PKCδ and PKCα respectively, are sequentially up-regulated by FGFR2b signaling

**Fig. 6:**
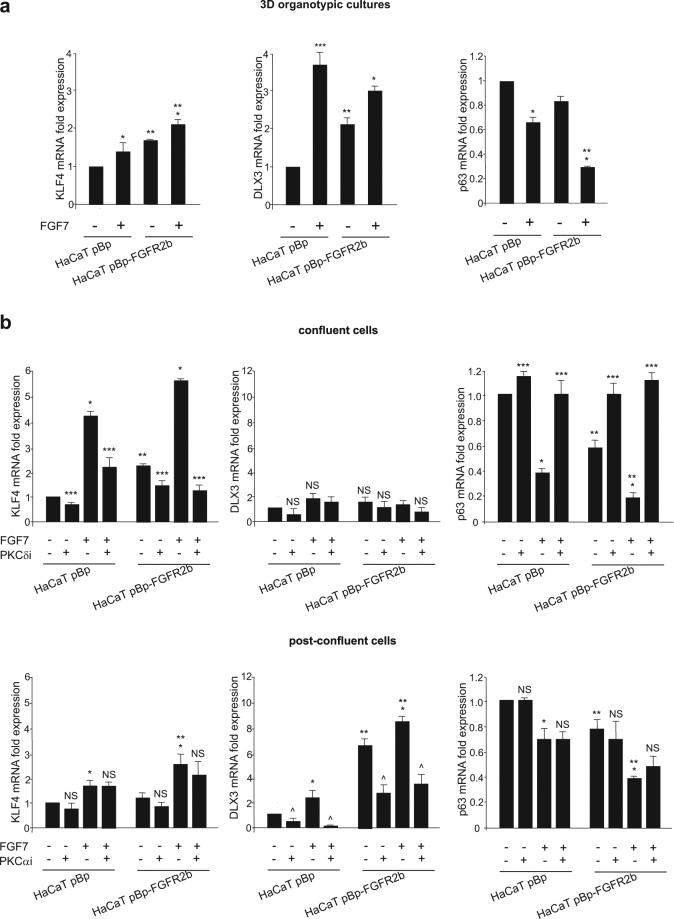
PKCδ and PKCα signaling are responsible for FGF7-mediated sequential induction of KLF4 and DLX3, respectively. **a** Organotypic skin equivalents of HaCaT pBp and pBp-FGFR2b clones, prepared as reported in Materials and methods section, were grown in complete medium and left untreated or stimulated with FGF7 as above. Real time RT-PCR analysis shows a significant increase of both KLF4 and DLX3 expression upon FGF7 stimulation, while p63 displays an opposite trend. **b** HaCaT pBp and HaCaT pBp-FGFR2b cells were grown up to confluence or post-confluence in the presence or not of PKCδ and PKCα inhibitors, respectively, and stimulated with FGF7 as above. Real time RT-PCR analysis shows that KLF4 up-regulation is mainly appreciable in confluent clones, while DLX3 induction is detectable only in post-confluent cultures. The increased expression of KLF4 in confluent cells and that of DLX3 in post-confluent cultures are significantly dampened by PKCδi and PKCαi, respectively. Similarly to KLF4, p63 down-modulation in response to FGF7 is more evident in confluent cells and it appears impaired only by PKCδi. Results are expressed as mean values ± SD. Student’s *t* test was performed and significance levels have been defined as above: **a** *p < 0.05 and ***p < 0.001 vs the corresponding FGF7-unstimulated cells; **p < 0.05 vs the corresponding pBp cells; **b** NS and *p < 0.05 vs the corresponding FGF7-unstimulated cells; NS and **p < 0.05 vs the corresponding pBp cells; NS and ***p < 0.05 vs the corresponding PKCδ inhibitor-untreated cells; NS and ^*p* < 0.005 vs the corresponding PKCα inhibitor-untreated cells

Since it has been recently proposed that PKCδ and PKCα control the expression of several genes via the induction of KLF4^28,29^ and DLX3^36^ respectively, we decided to investigate the possible involvement of these transcription factors in FGFR2b-triggered keratinocyte differentiation. The topic was particularly appealing, since they have been proposed to be involved in an intricate network of loops with ΔNp63^47–49^, the transcription factor whose down-modulation represents the main molecular mechanism known to drive differentiation of stratified epithelia^17^. Indeed, we have already described a link between FGFR2b and p63^16^, but the molecular mechanism underlying this interplay remains to be still clarified. To this aim, we performed Real Time RT-PCR analysis on HaCaT pBp and pBp-FGFR2b 3D organotypic rafts, checking for the transcript levels of KLF4 and DLX3 and comparing them with that of p63. The results showed a significant increase of both KLF4 and DLX3 and an opposite decrease of p63 in response to FGF7 stimulation (Fig. 6a), which were more evident in the rafts overexpressing FGFR2b (Fig. 6a).

Then, to verify if KLF4 and DLX3 up-regulation could display a sequential trend during FGFR2b-induced keratinocyte differentiation, we analyzed their possible modulation in confluent and post-confluent clones stimulates with FGF7 as above. The results showed that KLF4 up-regulation was mainly appreciable in confluent clones (Fig. 6b), while DLX3 induction was detectable only in post-confluent cultures (Fig. 6b). As expected, the induction of KLF4 in confluent cells as well as that of DLX3 in post-confluent cells were significantly counteracted by PKCδi and PKCαi, respectively (Fig. 6b), further confirming the connection of each PKC signaling with the corresponding transcription factor. Moreover, similarly to what observed for KLF4 and consistently with its well-established role in FGFR2b-induced onset of differentiation^15,16^, p63 repression in response to FGF7 was more evident in confluent cells and impaired only by PKCδi (Fig. 6b). Being p63 a direct negative regulator of KLF4 in normal human keratinocytes^49^, its strong repression observed in confluent cells may represent the molecular mechanism through which FGFR2b-triggered PKCδ signaling would induce KLF4 and consequently the onset of keratinocyte differentiation. These results suggest that the alternative induction of KLF4 and DLX3 in FGF7-stimulated confluent and post-confluent cells, respectively, might reflect the sequential activation of their upstream activators PKCδ and PKCα during the early and late steps of FGFR2b-induced differentiation.

## Discussion

Because of its hypothesized role as a tumor suppressor in vitro and in vivo^9,10^, forced modulation of FGFR2b could be a winning strategy to revert important aspects of the malignant phenotype in carcinoma cells, including loss of cell differentiation. However, in the physiological context of normal human keratinocytes, despite the numerous evidences emphasizing the role of FGFR2b in the enhancement of early differentiation, only few and contrasting reports appear to suggest its possible involvement in the later stages of this process^18–20^. Therefore, here we first analyzed the impact of the receptor signaling on the induction of terminal differentiation in 3D organotypic rafts and 2D cultures of human keratinocytes engineered to stably overexpress FGFR2b. The results obtained clearly indicated that, particularly when FGFR2b is overexpressed, the receptor signaling not only induces a precocious onset of the early differentiation markers, but also enhances the expression and accelerates the appearance of the terminal markers. These findings strongly suggest a key role of FGFR2b in the regulation of the entire differentiation program.

Then, in order to analyze the signaling players downstream FGFR2b possibly involved in keratinocyte differentiation, we focused on PKCδ and PKCα isoforms, at the light of previous evidences showing their sequential role in the regulation of the different steps of the process^23^. To the aim to “dissect” in vitro the differentiation steps, cells were grown until confluence or up to post-confluence. Taking advantage of the use of specific substrate inhibitors and siRNA interference, we highlighted that PKCδ signaling appears to drive the early molecular events, while PKCα regulates the late ones, of FGFR2b-triggered keratinocyte differentiation. Given the role of PKCδ in the onset of the process, we used the organotypic cultures to analyze the impact of its inhibition on the ability of keratinocytes to terminally differentiate and stratify in response to ligand-mediated activation of FGFR2b: we demonstrated that PKCδ signaling shut-off was sufficient to block the entire differentiation program, inducing retention of undifferentiated/basal features and defective stratification. Interestingly, these rafts also maintained a sustained cell proliferation throughout all the layers that could be explained by the previously described ability of FGFR2b to not only promote the differentiation program^15^, but also sustain the residual proliferative activity of suprabasal, differentiating keratinocytes^14^. Overall, the phenotypic features derived by PKCδ signaling shut-off indicate that the inhibition of this signaling pathway alone is sufficient to impair the balance between cell proliferation and differentiation controlled by FGFR2b. On the other hand, we can not exclude the contribution of the epidermal growth factor receptor (EGFR) in such increased cell proliferation: in fact, the antagonistic role of FGFR2b and EGFR in epithelial cells has been previously described by our group^50^. While FGFR2b is up-regulated during the shift from the basal to the suprabasal layers, EGFR mainly displays a basal distribution and its ligand-dependent activation triggers cell proliferation. In addition, it has been also proposed that FGF7/KGF treatment may induce endogenous synthesis of the EGFR ligand TGFα.^51^ In any case, these results are in agreement with the widely described role of PKCδ as tumor suppressor, which is down-regulated in squamous cell carcinomas (SCCs)^52,53^.

Concerning the molecular effectors acting downstream PKC isoforms, our results showed that, in agreement with previous reports^28,29,36^, PKCδ and PKCα isoforms act via the induction of the downstream transcription factors KLF4 and DLX3, respectively. In fact, the expression of both KLF4 and DLX3 was clearly induced in response to FGF7, particularly in rafts or cell cultures overexpressing FGFR2b. Interestingly, in cultures grown at different cell densities to mimic the various steps of differentiation, we found that KLF4 up-regulation was mainly appreciable in confluent cells, while DLX3 induction was detectable only in post-confluent cultures. These sequential trends of induction could reflect the progressive involvement of their upstream inductors PKCδ and PKCα in the distinct stages (early and late, respectively) of differentiation.

Finally, we also decided to compare the modulation of KLF4 and DLX3 with that previously described for p63 and induced by FGFR2b signaling^16,41^. In fact, the FGFR2b/p63 crosstalk appears to be central in the homeostatic balance between keratinocyte proliferation and differentiation and its impairment, due to out-of-context appearance of FGFR2c, leads to loss of the differentiated phenotype and acquisition of tumorigenic features^41^. For this reason, to identify the molecular players contributing to the regulation of this crosstalk seems to represent a very urgent and important goal. Indeed, both KLF4 and DLX3 have been proposed to be involved in negative loops with p63:^48,49^ consistent with these assumptions, we observed that they displayed opposite modulations compared to p63. Interestingly, the up-regulation of KLF4 appeared more appreciable in confluent cells, the same culture condition in which the down-regulation of p63 was more evident: this simultaneous detection of p63 down-regulation and KLF4 induction mainly in confluent cells strongly suggests that they take place in the same step of the differentiation program, and possibly during the shift between the basal to the suprabasal layer. These observations, together with the recent evidence of a possible direct role of PKCδ in p63 down-regulation^54^, allowed us to speculate that the down-regulation of p63 could represent the molecular mechanism through which FGFR2b and its downstream PKCδ signaling could induce the up-regulation of KLF4 and consequently the onset of keratinocyte differentiation.

## Materials and methods

### Cells and treatments

The human keratinocyte cell line HaCaT^40^, stably overexpressing FGFR2b (pBp-FGFR2b) or the empty vector (pBp) and generated as previously described^6^, were cultured in Dulbecco’s modified Eagle’s medium (DMEM), supplemented with 10% fetal bovine serum (FBS) plus antibiotics. Primary cultures of human fibroblasts derived from healthy skin (HFs) were obtained from patients attending the Dermatology Unit of the Sant’Andrea Hospital of Rome; all patients were extensively informed and their consent for the investigation was given and collected in written form in accordance with guidelines approved by the management of the Sant’Andrea Hospital. HFs were isolated and cultured as previously described^55^.

For growth factors stimulation, cells were left untreated or incubated with FGF7 (Upstate Biotechnology, Lake Placid, NY, USA, 01–118) 100 ng/ml for 24 or 48 h at 37 °C. To inhibit AKT or MEK1/2 or PKCδ or PKCα cells were respectively incubated with the specific AKT inhibitor 1L-6-hydroxy-methyl-chiro-inositol 2-(R)-2-O-methyl-3-O-octadecylcarbonate (1 μM; Calbiochem, Nottingham, UK, 124005) or with the specific MEK1/2 inhibitor PD0325901 (1 μM; Sigma-Aldrich, Saint Louis, MO, USA, PZ0162) or with the specific PKCδ inhibitor rottlerin (5 μM; Calbiochem, 557370) or with the specific PKCα inhibitor Go6976 (3 μM; Calbiochem, 365250) for 1 h at 37 °C before treatment with FGF7 in the presence of each inhibitor.

For RNA interference and PKCδ or PKCα silencing, cells were transfected with PKCδ small interfering RNA (PKCδ siRNA) (Santa Cruz Biotechnology, Santa Cruz, CA, USA), or PKCα small interfering RNA (PKCα siRNA) (Santa Cruz Biotechnology), or with an unrelated siRNA as a control (control siRNA), using Lipofectamine 2000 Transfection Reagent (Invitrogen, Carlsbad, CA, USA) according to the manufacturer’s protocol.

### Organotypic cultures

For three-dimensional (3D) organotypic cultures, collagen rafts were prepared adding 5 mg/ml rat tail type I collagen (Corning, Lowell, MA, USA) to DMEM and Reconstitution buffer (8:1:1) as previously described^56^. HFs (1 × 10^6^) were added to 2 ml of the collagen mixture in polycarbonate micron inserts (23 mm diameter, pore size 0.3 µm; Corning) in 6-deep well plates (Corning). The mixture was left to polymerize for 30 min at 37 °C. After 24 h 2 × 10^5^ HaCaT pBp or pBp-FGFR2b cells were seeded on the collagen gel and left to grow for a week in complete medium added in both the top and the bottom wells. Then, the organotypic cultures were lifted to the air–liquid interface and cultured for further two weeks in complete medium added or not with the specific PKCδ inhibitor rottlerin (5 μM; Calbiochem, 557370). Then cultures were left untreated or stimulated with FGF7 (Upstate Biotechnology) 100 ng/ml for the last 4 days. Rafts were finally fixed in 10% formalin, embedded in paraffin and 4 µm slices were obtained. To evaluate organotypic thickness slices were stained with hematoxylin and eosin using standard procedures then bright field images were taken with an Axiocam ICc 5 (Zeiss, Oberkochen, Germany) connected with an Axioplan 100 microscope (Zeiss). The organotypic culture thickness was measured using the Axiovision software (Zeiss) and expressed as mean µm ± standard deviation (SD). Student’s *t* test was performed and significance levels have been defined as *p* < 0.05.

### Immunofluorescence

HaCaT clones, grown on coverslips, were fixed with 4% paraformaldehyde in PBS for 30 min at 25 °C followed by treatment with 0.1 M glycine for 20 min at 25 °C and with 0.1% Triton X-100 for additional 5 min at 25 °C to allow permeabilization. Cells were then incubated for 1 h at 25 °C with the following primary antibodies: rabbit polyclonal anti-K1 (1:50 in PBS, AF 87, Covance, Princeton, NJ, USA) or mouse monoclonal anti-β1 integrin (1:1000 in PBS; ST2–16, Santa Cruz Biotechnology). The primary antibodies were visualized using goat anti-rabbit IgG-Texas Red (1:200 in PBS; Jackson Immunoresearch Laboratories, West Grove, PA, USA) or goat anti-mouse IgG-Alexa Fluor 488 (1:200 in PBS; Life Technologies, Carlsbad, CA, USA) antibodies for 30 min at 25 °C. Nuclei were stained with DAPI (1:1000 in PBS; Sigma). Coverslips were finally mounted with mowiol (Sigma) for observation.

Organotypic raft sections, obtained as above, were deparaffinized in xylene and re-hydrated through graded ethanols to PBS, pH 7.4. Antigen retrieval was achieved by heating sections in target retrieval solution low pH (Dako, Carpinteria, CA, USA) for 15 min at 97 °C. Sections were then washed with PBS and blocked using 10% bovine calf serum and 0.2% Triton X-100 for 30 min at 25 °C before staining with rabbit polyclonal anti-K1 (1:500 in PBS, AF 87, Covance), mouse monoclonal anti-filaggrin (1:1000 in PBS, 15C10, Monosan Sanbio, Uden, The Netherland), rabbit polyclonal anti-cytokeratin 5 (1:400 in PBS, Abcam, Cambridge, UK) or mouse monoclonal anti-Ki67 (1:100 in PBS, MIB-1, Dako) antibodies for 1 h in a humidified chamber. The primary antibodies were visualized using goat anti-mouse IgG-Texas Red (1:200 in PBS; Jackson Immunoresearch Laboratories) or goat anti-rabbit IgG-Texas Red (1:200 in PBS; Jackson Immunoresearch Laboratories) antibodies for 30 min at 25 °C. Nuclei were stained with DAPI (1:1000 in PBS; Sigma). Sections were permanently mounted under a coverslip. Fluorescence signals were analyzed by conventional fluorescence or by scanning cells in a series of sequential sections with an ApoTome System (Zeiss) connected with an Axiovert 200 inverted microscope (Zeiss); image analysis was performed by the Axiovision software (Zeiss) and images were obtained by 3D reconstruction of the total number of the serial optical sections. Quantitative analysis of the fluorescence intensity was performed by the Axiovision software (Zeiss), analyzing 10 different fields randomly taken from three independent experiments. Quantitative analysis of the percentage of Ki67 positive cells was assessed counting for each sample a total of 50 cells, randomly observed in 10 microscopic fields from three different experiments. Results are shown as means ± standard error (SE). Student’s *t* test was performed and significance levels have been defined as *p* < 0.05.

### Western blot analysis

Cells were lysed in a buffer containing 50 mM HEPES, pH 7.5, 150 mM NaCl, 1% glycerol, 1% Triton X-100, 1.5 mM MgCl_2_, 5 mM EGTA, supplemented with protease inhibitors (10 μg/ml aprotinin, 1 mM PMSF, 10 μg/ml leupeptin), and phosphatase inhibitors (1 mM sodium orthovanadate, 20 mM sodium pyrophosphate, 0.5 M NaF). A range between 20 and 50 μg of total protein was resolved under reducing conditions by 8 or 12% SDS-PAGE and transferred to reinforced nitrocellulose (BA-S 83, Schleider and Schuell, Keene, NH, USA). The membranes were blocked with 5% nonfat dry milk in PBS 0.1% Tween 20 and incubated with anti-DSG1 (27B2, Life Technologies), anti-β1 integrin (ST2–16, Santa Cruz Biotechnology), anti-involucrin (SY5, Abcam), anti-filaggrin (15C10, Monosan), anti-p-PKCα (Ser657, Abcam) monoclonal antibody or with anti-K1 (AF 87, Covance), anti-Bek (C-17, Santa Cruz Biotechnology), anti-loricrin (Covance), anti-p-PKCδ (Ser645, Santa Cruz Biotechnology) polyclonal antibodies, all followed by enhanced chemiluminescence detection (ECL, Amersham, Alington Heights, IL, USA). The membranes were rehydrated by being washed in PBS-Tween 20, stripped with 100 mM mercaptoethanol and 2% SDS for 30 min at 55 °C, and probed again with anti-PKCδ (C-20, Santa Cruz Biotechnology) polyclonal antibodies, anti-PKCα monoclonal antibody (Y124, Abcam) or with anti-β-ACTIN (AC-15, Sigma) monoclonal antibody or anti-α-TUBULIN (2148S, Cell Signaling) polyclonal antibodies to estimate the protein equal loading. Densitometric analysis was performed using Quantity One Program (Bio-Rad Laboratories, Hercules, CA, USA). Results from three different experiments were normalized and expressed as fold increase respect to the control value. Values from a representative of three independent experiments were reported in each figure.

### Primers

Oligonucleotide primers necessary for target genes and the housekeeping gene were chosen utilizing the online tool Primer-BLAST and purchased from Invitrogen. Primers list and characteristics are reported in Supplementary Table S1. For *p63* target gene, the reported primers were designed to recognize both ΔNp63 and TAp63 α and β isoforms. For each primer pair, we performed no-template control and no-reverse-transcriptase control (RT negative) assays, which produced negligible signals.

### RNA extraction and cDNA synthesis

Organotypic cultures were deparaffinized and RNA was extracted using the TRIzol method (Invitrogen) according to manufacturer’s instructions and eluted with 0.1% diethylpyrocarbonate (DEPC)-treated water. Each sample was treated with DNAase I (Invitrogen). Total RNA concentration was quantitated by spectrophotometry; 1 μg of total RNA was used to reverse transcription using iScriptTM cDNA synthesis kit (Bio-Rad) according to manufacturer’s instructions.

### PCR amplification and real time quantitation

Real time RT-PCR was performed using the iCycler Real Time Detection System (iQ5 Bio-Rad) with optimized PCR conditions. The reaction was carried out in 96-well plate using iQ SYBR Green Supermix (Bio- Rad) adding forward and reverse primers for each gene and 1 μl of diluted template cDNA to a final reaction volume of 15 μl. All assays included a negative control and were replicated three times. The thermal cycling program was performed as described^6^. Real time quantitation was performed with the help of the iCycler IQ optical system software version 3.0a (Bio-Rad), according to the manufacturer’s manual. Results are reported as mean ± SD from three different experiments in triplicate. Student’s *t* test was performed and significance levels have been defined as *p* < 0.05.

### Statistical analysis

Data were statistically analyzed with unpaired two-tailed Student’s *t* test. Differences were considered significant at the level of *p* < 0.05. Statistical analysis was performed by using Microsoft Excel 2016 Spreadsheet Software (Microsoft® Software; Redmond, Washington, United States).

## Electronic supplementary material

Supplementary Table S1

Supplementary Figure S1

Supplementary Figure S2
