# Supplementary material for: Role of FGFR2b expression and signaling in keratinocyte differentiation: sequential involvement of PKCδ and PKCα
Source: Cell Death Dis. 2018 May 11;9(5):565. doi: 10.1038/s41419-018-0509-x (PMC5948219; doi:10.1038/s41419-018-0509-x)
Supplement: Supplementary file 1 — Supplementary Table S1 [file 41419_2018_509_MOESM1_ESM.pdf]

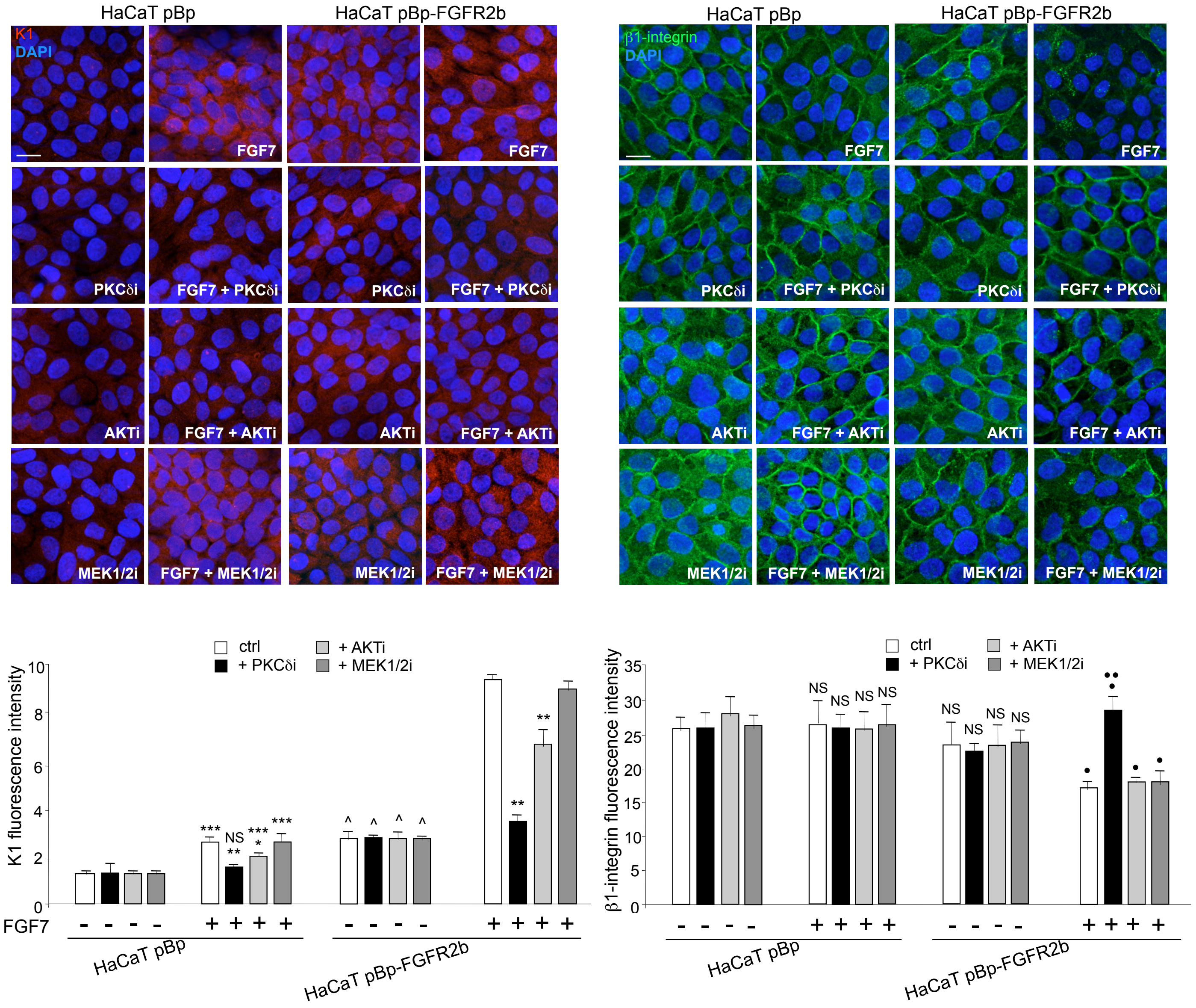

Supplementary Figure S2. PKCδ is the hub signaling substrate regulating the entire FGFR2b-induced early differentiation, while AKT function appears restricted to some aspects of the process. HaCaT pBp and HaCaT pBp-FGFR2b grown up to confluence were left untreated or stimulated with FGF7 in presence or not of the indicated substrate inhibitors as reported in Materials and methods. Quantitative immunofluorescence analysis shows that, particularly in pBp-FGFR2b cells, PKCδ inhibitor interferes with the increase of K1 signal and with the repression of β1-integrin staining induced by FGF7 stimulation (left and right panels, respectively). In addition, AKT inhibitor interferes only with FGF7-induced up-modulation of K1, but not on the FGF7-mediated reduction of β1-integrin (left and right panels, respectively). MEK1/2 inhibitor results always ineffective (left and right panels). Quantitative analysis of the fluorescence intensity was performed as described in Materials and methods and results are expressed as mean values ± SE. Student's t test was performed and significance levels have been defined as above: *$p < 0.01$, **$p < 0.0001$ and ••$p < 0.001$ vs the corresponding control cells; NS, ***$p < 0.0001$ and •$p < 0.05$ vs the corresponding FGF7-unstimulated cells; NS and ^$p < 0.0001$ vs the corresponding pBp cells. Bar: 10 μm.
