# Supplementary material for: Role of FGFR2b expression and signaling in keratinocyte differentiation: sequential involvement of PKCδ and PKCα
Source: Cell Death Dis. 2018 May 11;9(5):565. doi: 10.1038/s41419-018-0509-x (PMC5948219; doi:10.1038/s41419-018-0509-x)
Supplement: Supplementary file 2 — Supplementary Figure S1 [file 41419_2018_509_MOESM2_ESM.pdf]

a

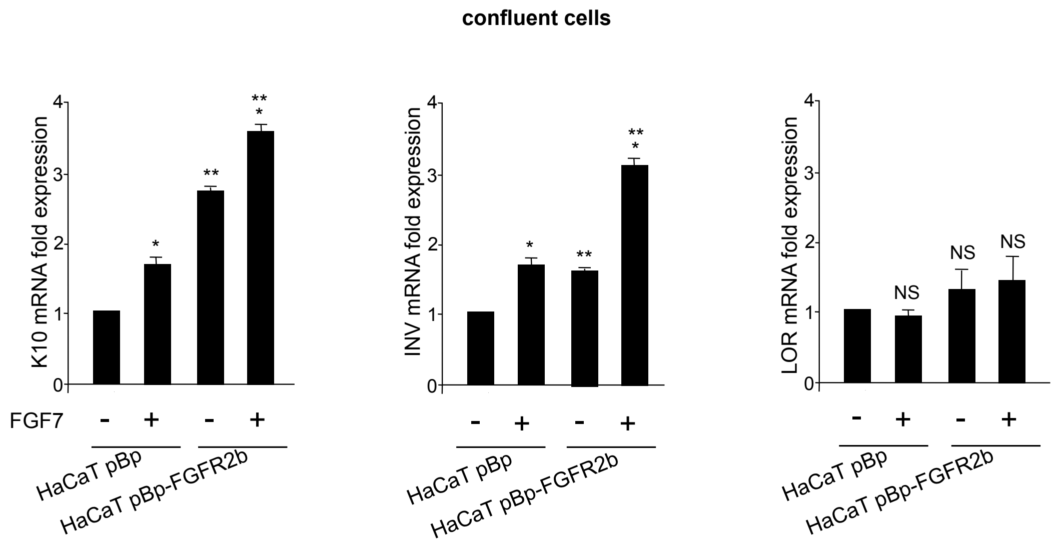

b

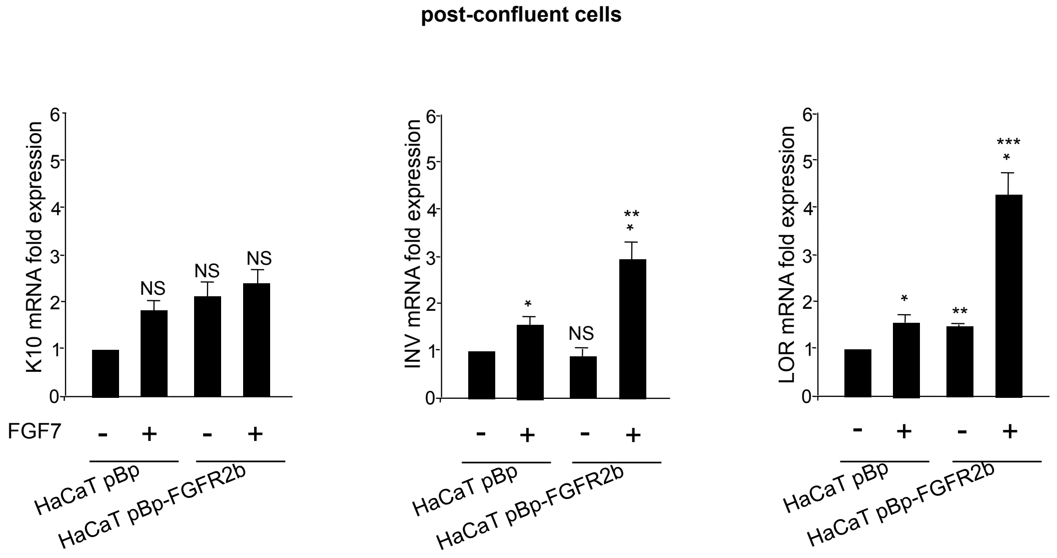

Supplementary Figure S1. Early/intermediate and late differentiation marker genes are sequentially induced by FGFR2b signaling in keratinocytes. HaCaT pBp and HaCaT pBp-FGFR2b clones grown up to confluence or post-confluence were left untreated or stimulated with FGF7 as reported in Materials and methods. (**a** and **b**) Real Time RT-PCR analysis shows that mRNA expression of K10 and INV increases upon FGF7 stimulation particularly in pBp-FGFR2b clones in confluent cells, while their modulation appears less evident in post-confluent cells. In addition, LOR expression appears up-regulated in response to FGF7 stimulation, particularly in cells overexpressing FGFR2b, only in post-confluent conditions. Results are expressed as mean values ± SD. Student's t test was performed and significance levels have been defined as above: (**a**) NS and *$p < 0.05$ vs the corresponding FGF7-unstimulated cells; NS and **$p < 0.05$ vs the corresponding pBp cells (**b**) NS and *$p < 0.05$ vs the corresponding FGF7-unstimulated cells; NS, **$p < 0.05$ and ***$p < 0.005$ vs the corresponding pBp cells.
